# Coronary Artery Bypass Surgery in a Preoperative Total Laryngectomy Patient

**DOI:** 10.7759/cureus.36610

**Published:** 2023-03-23

**Authors:** Emeka B Kesieme, Erica Tsoi, Keith G Buchan

**Affiliations:** 1 Cardiothoracic Surgery, Aberdeen Royal Infirmary, Aberdeen, GBR; 2 Medicine and Surgery, University of Aberdeen School of Medicine, Aberdeen, GBR

**Keywords:** permanent tracheostomy, recurrent laryngeal carcinoma, laryngectomy, coronary artery bypass grafting, manubrium-sparing sternotomy

## Abstract

For patients awaiting urgent total laryngectomy who require coronary artery bypass grafting (CABG), the conventional median sternotomy should be avoided. We present a 69-year-old male who had urgent CABG as a prelude to an urgent laryngectomy for recurring laryngeal carcinoma. We recommend a manubrium-sparing T-shaped ministernotomy to preserve tissues and to avoid the disruption of the anatomy of the lower neck and superior mediastinum.

## Introduction

Numerous papers have been published dealing with the topic of how to undertake cardiac surgery in patients who have previously undergone laryngectomy [1-3]. We have been unable to find any article dealing with the rarer situation of someone awaiting laryngectomy who required urgent cardiac surgery as a prelude to major head and neck surgery. It occurred to us that a manubrium-sparing sternotomy would allow for earlier mobilisation in someone with airway compromise who could be expected to struggle with expectoration postoperatively. Additionally, the lack of a surgical incision in the territory of the anticipated laryngectomy would be advantageous.

Manubrium-sparing sternotomy has been described before in the context of minimal-access cardiac surgery [4] and undertaking cardiac surgery in someone with a permanent tracheostomy [1].

## Case presentation

A 69-year-old male, a known patient of otolaryngology, was admitted into the coronary care unit with severe chest tightness, which was non-radiating. ECG revealed subtle inferior changes. Troponin was not elevated. Coronary angiogram revealed moderate plaque in the distal left main stem (LMS), severe stenosis of the proximal and middle left anterior descending (LAD) artery, severe stenosis of the left circumflex (LCX) artery and severe stenosis of the middle right coronary artery (RCA) and posterior descending artery (PDA). The Agatston calcium score was 763 on cardiac CT angiogram (LMS, 94; LAD, 493; LCX, 84; and RCA, 92). Echocardiography showed mild left ventricular impairment with midapical, anterolateral and posterolateral regional wall motion abnormality.

**Figure 1:**
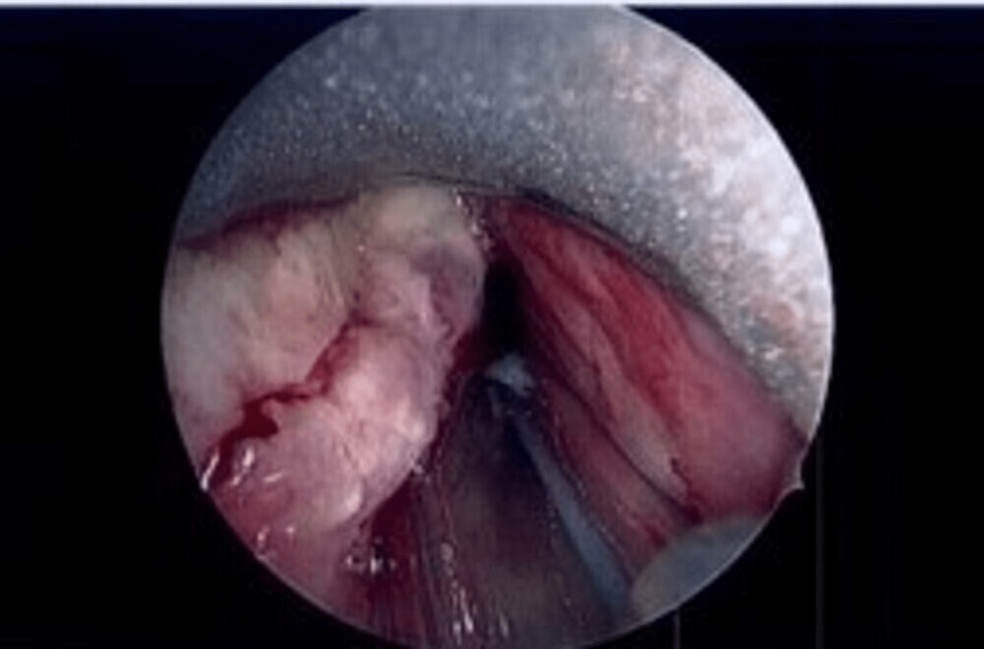
Tumour in the left posterior glottic region

He had a history of laryngeal carcinoma (T1a squamous cell carcinoma of the left vocal cord), which was treated with radical radiotherapy three years previously. He started experiencing a recurrence of hoarseness. Laryngoscopy was performed, which confirmed the recurrence of laryngeal cancer affecting the left posterior glottic region with extension into the paralaryngeal fat, and biopsy was taken (Figure 1).

CT scan confirmed T3 N0 M0, and the tumour was assessed as suitable for salvage total laryngectomy and neck dissection. After a discussion between the members of the multidisciplinary team (MDT), it was agreed that the best course of action would be to carry out coronary artery bypass grafting (CABG) before laryngectomy.

**Figure 2:**
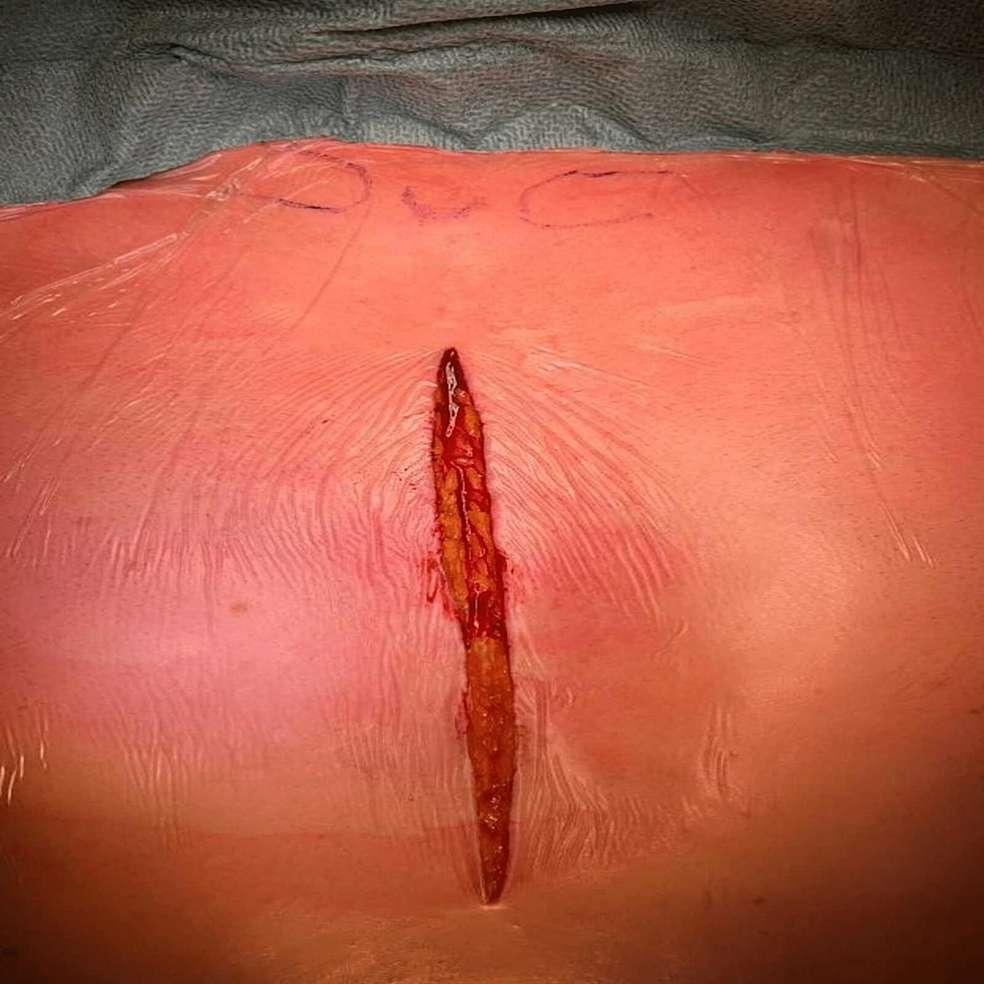
Image showing planned skin incision, medial end of both clavicles and marked sternal notch

After skin preparation, we marked the sternal notch and both medial ends of the clavicle and took note of the sternal angle of Louis (Figure 2).

**Figure 3:**
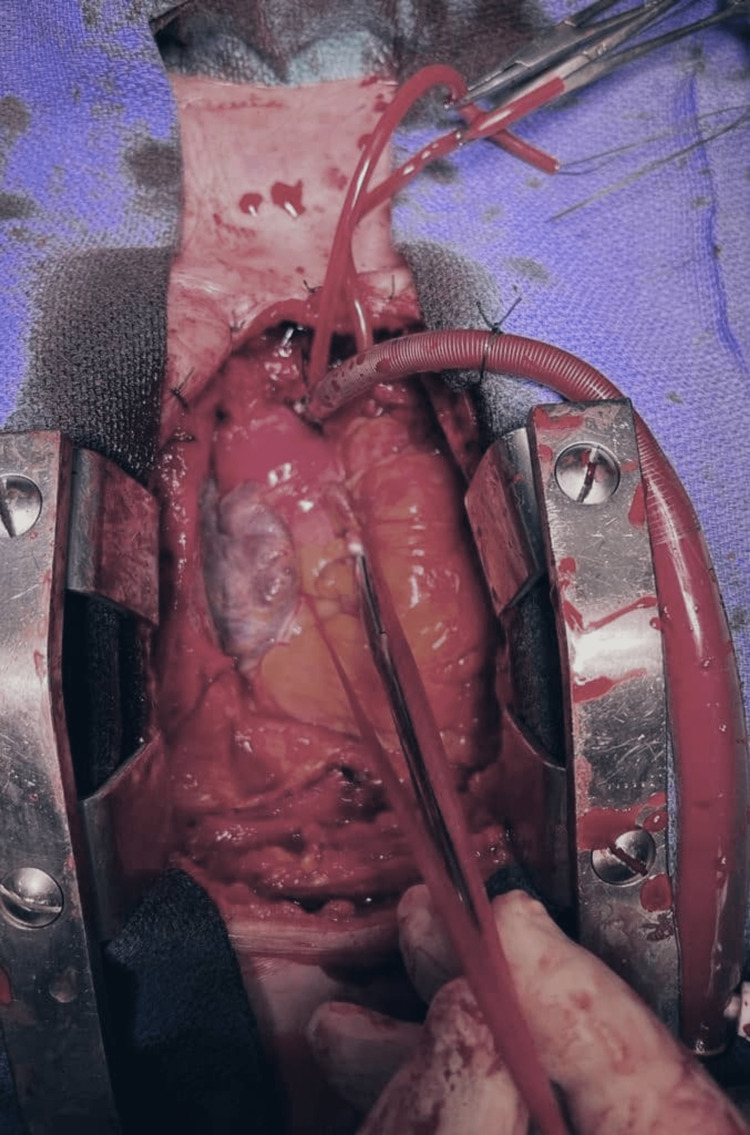
Manubrium-sparing sternotomy sparing the upper 6 cm

A midline skin incision was made 6-7 cm below the sternal notch to 2 cm below the xiphoid process. A manubrium-sparing sternotomy (T-shaped) was made using the oscillating saw to make a transverse cut at the level of first intercostal space. The upper 6 cm of the sternum was left intact (Figure 3).

The pericardium was opened, and the upper end was suspended to expose the right atrium and the ascending aorta. Aorto-atrial cannulation was carried out for cardiopulmonary bypass. He had vein graft to the left anterior descending (LAD) artery, obtuse marginal (OM) artery and posterior descending artery (PDA). The procedure was performed on-pump using blood cardioplegia. The bypass time was 99 minutes, whereas the cross-clamp time was 48 minutes. Two chest drains were positioned in the right pleura and the mediastinum. The sternum was approximated with figure-of-eight heavy stainless steel wires. The skin and superficial tissues were closed in layers. He made an uneventful recovery after cardiac surgery and was transferred to the otolaryngologists.

He subsequently had salvage laryngectomy and bilateral selective neck dissection two months after CABG. Check laryngoscopy revealed the resectable tumour of the left larynx. Apron incision was used, and stoma incision was made in the lower skin flap. The removed larynx, left neck dissection (levels 2-3) and right neck dissection (levels 2-4) were all sent for histology. He made remarkable improvement post laryngectomy.

## Discussion

Whilst the largest series of manubrium-sparing sternotomy cases recommended dividing the sternum transversely at the level of the first intercostal space, others have recommended doing so at the level of the second interspace [5-7]. We found the access in our case to be adequate, but there was a considerable restriction on the degree of retractor opening that was possible such that left-sided coronary grafting near the atrioventricular (AV) groove would have been rendered very difficult. Although the harvesting of the left internal mammary artery has been described with this approach, it would not be possible to dissect the upper 6-8 cm of the vessel via this surgical approach, which made it less attractive to use as a coronary bypass conduit [1,6]. We would not recommend making the transverse sternal incision at the level of the second interspace in cases where aortocoronary anastomoses are planned.

The preservation of the manubrio-clavicular osteo-ligamentous arch will put the patient in a better position for early mobilisation following cardiac surgery [8]. It will permit a stronger cough through better chest wall stability and less pain [8]. For the patient awaiting urgent major head and neck cancer surgery, it leaves the root of the neck untouched by surgical scars so that all options remain open at the time of subsequent surgery including rare situations such as the need for mediastinal tracheostomy [9]. Although we have not found any papers advocating it, we would use this incision in the context of a paraplegic patient or a bilateral above-knee amputee who requires cardiac surgery as these patients are very dependent on upper body strength for mobility.

## Conclusions

Urgent coronary artery bypass grafting in a patient requiring urgent total laryngectomy may not be a common clinical scenario. In patients with laryngeal carcinoma who require coronary bypass operation, we advise manubrium-sparing T-shaped ministernotomy. This should be applicable to patients with other head and neck tumours who require coronary artery bypass grafting as a prelude to definite surgery.
